# Amalgam, and Its Proper Use

**Published:** 1869-12

**Authors:** H. L. Sage

**Affiliations:** Bridgeport, Conn.


### AMALGAM AND ITS PROPER USE.

BY H. L. SAGE, BRIDGEPORT, CONN.

Amalgam Fillings—when are they indicated, or when is the Dental practitioner justified in their use for filling teeth, is a pertinent question, though a hackneyed one, at this time, when the increased sales and the multiplied number of manufacturers of the article, show an enormous annual consumption—a writer in the *Dental Times* having estimated it at a half ton.

There would seem to be, owing to the small amount of time, labor and skill required for its insertion in *comparison with gold*, a great temptation to the minds of unscrupulous, indolent and unskillful operators to use it almost indiscriminately—even going so far as to recommend it in preference, as being *better* as well as cheaper. This is no exageration.

But, it is not my purpose to wage a war of words against the proper and legitimate use of amalgam, for, with the improvements which have been made in the qualities of the article by conscientious manufacturers, thus overcoming in a great measure, if not fully, some of the objectionable features which pertained to its employment in days gone by, such as liability to shrinkage, oxydation, etc., it holds an important place in the office of the Dentist, and in certain c ses may be rendered very useful, if not indispensable. As to its deleterious effects upon the organism, that is a question which has never been fully settled, the great controversy of 1844-'7 not affording any definite decision of the point in dispute, and that when the quality of the amalgams were much inferior to what they are at the present day, not taking into account the often rude and careless method of manipulation and introduction into the cavities.

When such great lights in the profession as Harris, on this side of the water, and Tomes on the other, have disagreed in regard to its effects, who shall decide the question? To quote Harris, "amalgam is the most pernicious material that has ever been employed for filling teeth. When used in considerable quantity it is apt to exert a deleterious effect upon the alveolo-dental membranes, gums, and other parts of the mouth. Several decided cases of salivation occasioned by its use, have fallen under my observation."

Says Tomes: "It has been argued that the mercury used in making the alloy will salivate the patient. I have never seen a case in which this result was produced, and think we may fairly conclude, that the instances are so extremely rare, that they need not influence our practice."

Having thus prefaced my subject, let me suggest, that the value or efficiency of almost any remedy or appliance may be lost by an improper, imprudent, and indiscriminate use, or in general terms, the abuse thereof; so the method of working, employing, or applying any article in itself valuable, may have everything to do with its usefulness; and upon this depends the successful or unsuccessful accomplishment of the ends to be attained.

This being true, *when* and *how* as related to the use of amalgam as a combination of metals for filling teeth, suggests many important inquiries and their answers:

And first, when are amalgam fillings indicated, and when are we, and when not, justified in their employment is a question, the decision of which rests upon many governing circumstances which can not be answered except upon general considerations—individual cases being controlled, or should be, by a conscientious consideration of duty—love to God and love to man—and what is desirable, a correct appreciation on the part of the patient of our mutual relations, and his consequent permission to carry out the dictates of conscience and of judgment—the former unperverted and the latter enlightened by every possible means which may be brought to bear upon it.

Amalgam, in its legitimate use, is a kind of *dernier* resort as a means of preservation for a class of teeth broken down in structure, and deprived by caries of the greater part of their dentine, and so frail as to come under the familiar appellation of "shells." We often see those that, having been filled or refilled, perhaps with gold, or tin, or amalgam, or oftener not at all, that are, owing to a previous imperfect operation, to neglect, to changes in the fluids of the mouth, or the character of the tooth structure as relating to its proportions of earthy or animal matter, from disease or other causes too numerous to mention, even were they apparent—so far gone that nothing will so kindly approximate to the frail and delicate walls of the cavity, and at the same time prove so durable as amalgam; and such teeth are not unfrequently filled with the expectation that a year or two, at the farthest, will prove the limit of their existence, while it is often the case, that they are thus rendered useful for five, perhaps ten years, and even longer.

Again, there is another class of teeth, whose "name is legion," *i. e.*, those that, from various causes, such as calcareous deposit about the necks, or the presence of foreign matters, may have become so involved with the adjacent and connecting tissues in disease, that their speedy loss becomes probable. Some are decayed, loose and partially protruded from their sockets, while others are in one sense elongated, but not loose. They are with, or without antagonizing ones, as the case may be. It may be desirable to preserve them as long as possible; perhaps the retention of a plate with artificial teeth may be dependent upon them, or old age, or the refusal of the patient to have them extracted, may render it best to fill them. It becomes a serious question, however, whether such teeth had not better be removed at once, for we usually find them associated with diseased gums, and perhaps proving a constant source of irritation and attendant evils. If they are to be filled, however, amalgam may be appropriately used, for generally they will not pay for the insertion of gold (keeping out of view its greater purity and innocuous qualities, as compared with the former), even if the force required in its introduction did not hasten their destruction.

Again, a cavity is sometimes so situated that none but the most patient and skillful can so become master of the situation as to insert a gold filling, perfectly impacted and adapted to the walls of the cavity, which necessitates a freedom from moisture or other impediments to its successful insertion. But though such cases are comparatively rare, they nevertheless come under our notice; and it were better then to introduce a *perfect* amalgam filling, than to fail in the attempt to insert one of gold, sufficiently so to insure the preservation of the tooth.

Some advocate the use of amalgam for deciduous teeth, but the oxychloride of zinc is, in my opinion, preferable, even though its renewal may become necessary.

The above designated cases are the only ones in which I can conceive that it were not *better*, if practicable, to employ gold in preference to amalgam—keeping out of view the cupidity, want of discrimination, poverty or inclinations of the patient, preventive of a wise carrying out of the promptings of our best judgment, rendering applicable in such cases the saying of the Apostles, "when I would do good, evil is present with me;" a slight transposition of the meaning, however, the *evil* being present in the person of the patient.

The practice which obtains with some Dentists, even in these days of progress, of filling cavities in the incisors and cuspids with amalgam, can not be too strongly deprecated. It is hardly possible to conceive of a case in which such practice would be admissible—but there are practitioners, incredible as it may seem, who have distributed such favors (?) in great abundance, as the once beautiful, but now unsightly structures attest—the genial and God-given smile only making the hideousness all the more apparent, the dark and discolored enamel in the front teeth of the young and interesting remaining a silent witness to condemn the authors of the miserable defacement.

Again, amalgam fillings are not indicated in the approximal cavities of the bicuspids and molars, especially of the former. There is no class of teeth (if we except the six-year molars) so difficult to preserve, or in which so many failures are every day apparent as the bicuspids. Hence, success depends upon the most thorough and suitable preparation of the cavity previous to the insertion of the filling, which should be of gold, and the most perfect manipulation, impactation, and adaptation possible, but the details of which it is not my present purpose to present.

An amalgam filling will do better in the grinding surface of a tooth than in the approximal or buccal, or lingual, and indeed, a cavity in the first situation is more favorable for the permanence of any metalic filling than it would be in any of the latter, but in this even, the amalgam ought not to be used, if possible, to influence the patient; for an operator who can not make a good filling of gold in a cavity opening upon the grinding surface, will certainly fail if he attempts it in any other location, this being the most simple, and easy of access. If overruled in opposition to convictions of duty and the good of the patient, better yield to his peculiarities or circumstances, than that the teeth should be neglected and lost.

For example: A calls to have his daughter's teeth examined, and if necessary, filled. He "wishes you to distinctly understand that he is not going to pay the prices usually charged by Dentists," and wants "cheap fillings." "Amalgam is good enough—he has fillings of it, that were put in twenty years ago." The young lady is interesting, with a denture as regular and well developed as the average. You advocate the use of gold, and try to induce the father to submit to your superior judgment in the matter. You positively refuse to fill the front teeth with the objectionable material—it has never been your practice, and if you are possessed of true manliness, it never will be. So you compromise the matter by using gold for the anterior teeth, and tin or amalgam for the others. The father is wealthy, but picayunish and little. You might have filled all of the cavities with gold, and *charged* for cheap fillings, but perhaps you were poor, your time was precious and belonged to more worthy patrons, and you could not afford to tax your powers of endurance by such a course, or deprive yourself of the compensation by refusing to operate at all, and permitting him to go to Dr. Slambang across the way, who does not hesitate to *stuff all* carious teeth with amalgam, indiscriminately, if requested so to do, and poorly at that.

When the patient can not *afford* gold, and the teeth are in such a condition that there is a reasonable prospect of arresting the decay, by the employment of a cheaper material, then we may be justified in the judicious use of amalgam (if tin will not answer a better purpose)—that is, in the cavities situated in the back teeth—unless our circumstances will permit us to give the benefit of the best material, without an adequate pecuniary compensation.

In filling the roots and pulp cavities of teeth, amalgam should not be used. There is no excuse for the practice, for if a cheap filling is to be inserted, there are other plastic materials more suitable, and free from the objections which pertain to the use of the former. Who has not observed even ordinary cavities, with amalgam in the bottom and gold inserted upon it? Yes, and when the patient declared that he ordered and paid for an all gold filling, and supposed it to be such. If you have not, do you live near New York City? In such places "extremes meet."

Having thus considered some of the conditions in which the introduction of amalgam fillings would be admissible and proper, and those in which they would not, let us pass to the second division of the subject, the *how* as pertaining to the use of amalgam, always supposing, if I may be allowed a repetition, that circumstances call imperatively for its adoption. In the first place, the preparation of cavities, though often different as regards shape, etc., from what is required for gold, ought to be quite as thorough. Though most cavities requiring amalgam, or rather those in which we must use it, nolens volens, come to us with their friable borders, we find it necessary to retain as much of the "shell" as possible, compatible with strength, or the filling will be liable to become loose and fall out, and besides it is best to conceal its character as much as may be. We often find these fillings wedged in, but with no attachment to the walls of the cavities—the effect of contraction, or a want of thoroughness in their insertion, and producing by their presence, irritation of the gums, or affording lodgment for foreign matters, and thus hastening the process of disintegration and decay.

When the borders of the cavity are exceedingly frail, or at all ragged, it is well to cut away to some extent, being careful to leave depth sufficient for the retention of the filling, When necessary to cut away the borders at all, a V shaped space should be left. There should always be some separation where this material is used for approximal cavities, for an amalgam filling should never infringe upon a neighboring tooth by a too near approximation.

Contour fillings of amalgam would not be advisable, in teeth so near the front as the bicuspids, owing to its unsightly appearance, and besides, their permanence would be endangered, which objection does not hold where gold is properly inserted.

We often observe, especially when approximal cavities in the bicuspids have been filled with this material, a contraction of the metal, as above referred to, or a drawing away from the substance of the tooth, one or both, and influenced by various causes, among which may be mentioned as probable —first, an imperfect preliminary cleaning out and shaping of the cavity; second, the absence of dentine to support the filling, as when the enamel only constitutes the borders, or quite a large extent of the surface of the cavity, the adhesion is not so perfect, owing probably to the great hardness and smoothness of enamel in comparison with dentine; third, the occasional fracture of the walls, if thin and delicate, an accident to which they are liable in mastication; fourth, the improper manner of mixing the material previous to its insertion; fifth, the careless and imperfect manner of packing it; sixth, the want of care on the part of the patient in not giving the filling sufficient time to harden before eating, thus disturbing its position and adhesion; or, seventh, the relative proportions of metals introduced and combined in its manufacture.

A cavity with overhanging borders, the orifices being smaller in diameter than the main or inside portions, is not objectionable, as regards the durability of the filling, where a plastic material is to be introduced, provided sufficient substance remains to insure strength; but, (excepting it may be necessary to leave a layer as a protection to a nearly exposed pulp) there should be a thorough removal of all carious matter, which sometimes extends even to the extreme points of the dentine in the cusps, and into the minute angles of tooth structure; and here again, special care should be exercised, so as to make these portions of the filling as solid and compact as possible.

Perhaps the use of the os-artificial in these angles, before filling over with amalgam, after leaving sufficient depth and proper shape for the retention of the latter, would be advisable inasmuch as not a little of the discoloration of the tooth would thus be prevented. Where there is a near approach of caries to the pulp it is certainly better to first introduce, after an application of creosote, a layer of the oxychloride of zinc, or of Hill's stopping, as a non-conducting material.

All such teeth can be filled with gold, with a reasonable prospect of permanence, and the natural shape and contour restored, by cutting away until firm borders are secured, and the use of suitable retaining points.

There is a class of teeth in which the retention of amalgam is somewhat difficult; for instance, where the walls are so broken down, say in a molar, as to involve a large portion of one of the approximal or buccal, and a part of the grinding surface, to such an extent as not to leave depth of cavity sufficient for the permanence of the filling.

Most have doubtless observed numerous failures in such cases, the amalgam soon breaking away from the walls, by the pressure brought to bear in mastication. Here again gold is much better, when it can be successfully introduced, but the most difficulty would be experienced in the inferior dentes-sapientiæ on the posterior or buccal surfaces.

If you have decided upon the use of amalgam where the security of the filling is doubtful, you will find *retaining points* very useful. By this means much more stability can be had and the tooth rendered useful much longer than otherwise. To contour fillings, of amalgam, in molar teeth, they greatly add permanence. It has been my practice to make use of them in the preparation of cavities for *amalgam* fillings, under the above circumstances, though not aware that others have done so. Let the suggestion rate at its true value.

In regard to the manipulation of amalgam, it is good practice, after a thorough mixture of the materials and washing in alcohol until all discoloration is removed, to introduce it in as dry a state as possible, consistent with its amalgamation, and also to bring to bear considerable force in its consolidation in the cavity, until the particles assimilate and assume a polished appearance.

A *minute* portion of the poison being left in the compound, it is certainly less objectionable on the score of its deleterious effects, if any there are, which is a mooted question, for it would seem that the mercury must necessarily remain in excess to produce injurious consequences. Again, if the above rules are observed, the fillings will retain their color and their contraction will be prevented. If it is introduced in a state so plastic that the fingers are the only instruments required (for fillings have been seen bearing their impress), it can not, it would seem, but exert a pernicious influence upon the health of the patient, especially if possessing a special susceptibility to its influence.

Another thing, the inflamed gums, consequent upon the overlapping or projection of the filling at the cervical portion of the cavity, is a trouble frequently noticed. There is too little regard paid to this matter, too much care can not be exercised in the consolidation and finish of the filling at this point, and if partially or wholly on the grinding surface, it should not be so full as to prevent the perfect occlusion of the teeth. Where two contiguous cavities are filled, if with different metals, like amalgam and gold, they should never be allowed to come in contact with each other, as mischief is sometimes manifested in such cases by the galvanic action produced. To conclude, some operators seem to think, judging by their practice, that no pains-taking is required in the preparation and introduction of an amalgam filling; but let such bear in mind that, "He that is faithful in that which is least, is faithful also in much; and he that is unjust in that which is least, is unjust also in much." Be faithful, be honest, be true.

Is it not to the slovenly, careless and indiscriminate manner of the employment of amalgam that we are to look, in a great measure, for the curses which rest and have rested upon it? When kept within prescribed and proper bounds it may serve a good purpose, though in comparison with gold, the cases are *few* in which the use of it should be adopted.

